# *Wolbachia*-mediated reproductive manipulation in rice planthoppers

**DOI:** 10.1007/s44297-025-00059-y

**Published:** 2025-10-23

**Authors:** Yue-Di Niu, Meng-Ke Wang, Zhi-Chao Yan, Xiao-Li Bing, Xiao-Yue Hong

**Affiliations:** Department of Entomology, Nanjing Agricultural University, Nanjing, 211800 Jiangsu China

**Keywords:** *Wolbachia*, Rice planthopper, Cytoplasmic incompatibility, Fecundity

## Abstract

Rice planthoppers, including brown (*Nilaparvata lugens*), small brown (*Laodelphax striatellus*), and white-backed (*Sogatella furcifera*) planthoppers, are major agricultural pests in China and severely affect rice production and food security. The endosymbiotic bacterium *Wolbachia* is commonly found in these insects, where it regulates reproduction through mechanisms such as cytoplasmic incompatibility (CI) and increased fertility. In this review, we discuss the strain-specific effects of *Wolbachia*: *w*Lug (in *N. lugens*, < 50% infection) increases fecundity without CI; *w*Stri (in *L. striatellus*, 99% infection) induces complete CI and enhances reproduction; and *w*Sfur (in *S. furcifera*, 90% infection) shows weak or no CI with minimal fecundity effects. Additionally, while *w*Stri can induce CI in *N. lugens*, its intensity is reduced, suggesting that both the symbiont and the host influence CI strength. The *w*Stri genome contains three copies of the CI factors *cifA-cifB*, which belong to a newly identified group of genes of unknown function. In *L. striatellus*, the host protein cytoplasmic aminopeptidase-like protein (CAL) is associated with CI lethality, whereas the NADH quinone oxidoreductase subunit A8 (NDUFA8) may play a role in CI "rescue". Furthermore, *Wolbachia* enhances rice planthopper reproduction through B vitamin synthesis, the upregulation of vitellogenin (Vg), and the promotion of germ cell division, significantly increasing egg production. These findings shed light on complex *Wolbachia*-planthopper interactions and their potential for pest control.

## Introduction

Rice planthoppers include three main pests: the brown planthopper *Nilaparvata lugens* (Stål), the small brown planthopper *Laodelphax striatellus* (Fallén) and the white-backed planthopper *Sogatella furcifera* (Horváth). Rice planthoppers damage rice plants by directly piercing and sucking, penetrating the stems by laying eggs, and transmitting rice diseases [1]. Although individuals are small, rice planthoppers have a high reproductive capacity, transmit numerous viruses, migrate over long distances, and are highly resistant to pesticides [2, 3]. They are the most widespread and extensively important crop pests [4]. Among the three rice planthoppers, in addition to the brown planthopper, which feeds exclusively on rice, the small brown planthopper and the white-backed planthopper also damage other crops. In particular, maize and wheat virus diseases transmitted by the small brown planthopper have experienced increasing outbreaks and epidemics in recent years, causing severe losses in agricultural production.

**Fig. 1:**
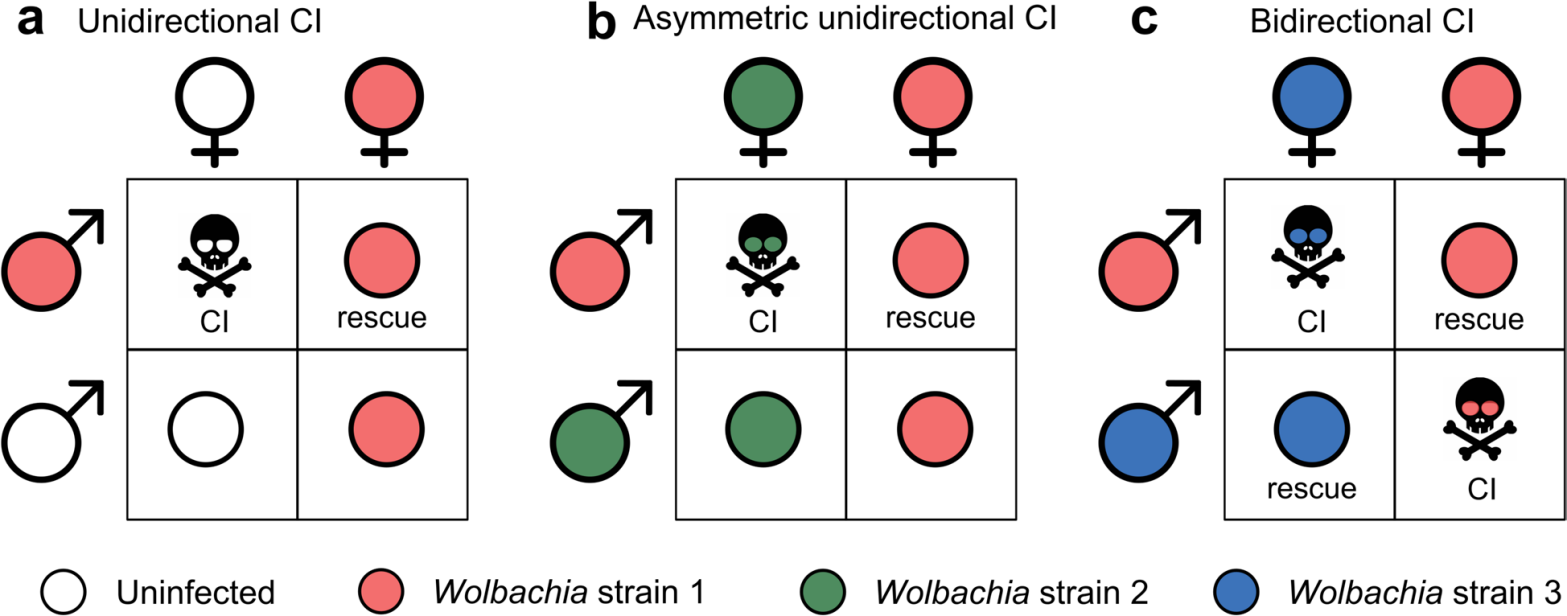
Types of *Wolbachia*-induced cytoplasmic incompatibility [8]. **a** Unidirectional CI, **b** asymmetric unidirectional CI, **c** bidirectional CI

*Wolbachia* is an intracellular symbiont that is widely distributed among insects and is capable of infecting reproductive tissues such as the testis and ovaries of the host. *Wolbachia* can be transmitted vertically to offspring through the maternal line [5, 6]. *Wolbachia* is known for regulating host reproduction, affecting host fecundity, and even regulating insect reproductive patterns and sex ratios. *Wolbachia* regulates host reproduction by killing infected males (male killing), causing genotypically male individuals to develop into females (feminization), producing females through parthenogenesis without mating or fertilization with a male (parthenogenesis), and inducing cytoplasmic incompatibility (CI) [7]. *Wolbachia*-induced CI refers to the phenomenon in which the offspring of fertilized eggs die during embryonic development after mating between a *Wolbachia*-infected male and an uninfected female (unidirectional cytoplasmic incompatibility) or a female infected with a different *Wolbachia* strain (bidirectional cytoplasmic incompatibility) (Fig. 1) [8]. These mechanisms can reduce or even kill males who do not directly help their transmission, increase the number of carrier females, and elevate the chance of bacterial transmission among host populations, which has important ecological significance.

This review focuses on the interaction between rice planthoppers and *Wolbachia* and elucidates the role and mechanisms of *Wolbachia* in regulating rice planthopper reproduction, which may contribute to uncovering the mechanisms of rice planthopper disasters and exploring new technologies for integrated rice planthopper control via symbiotic bacteria.

### Types of *Wolbachia*-mediated reproductive regulation in rice planthoppers

**Fig. 2:**
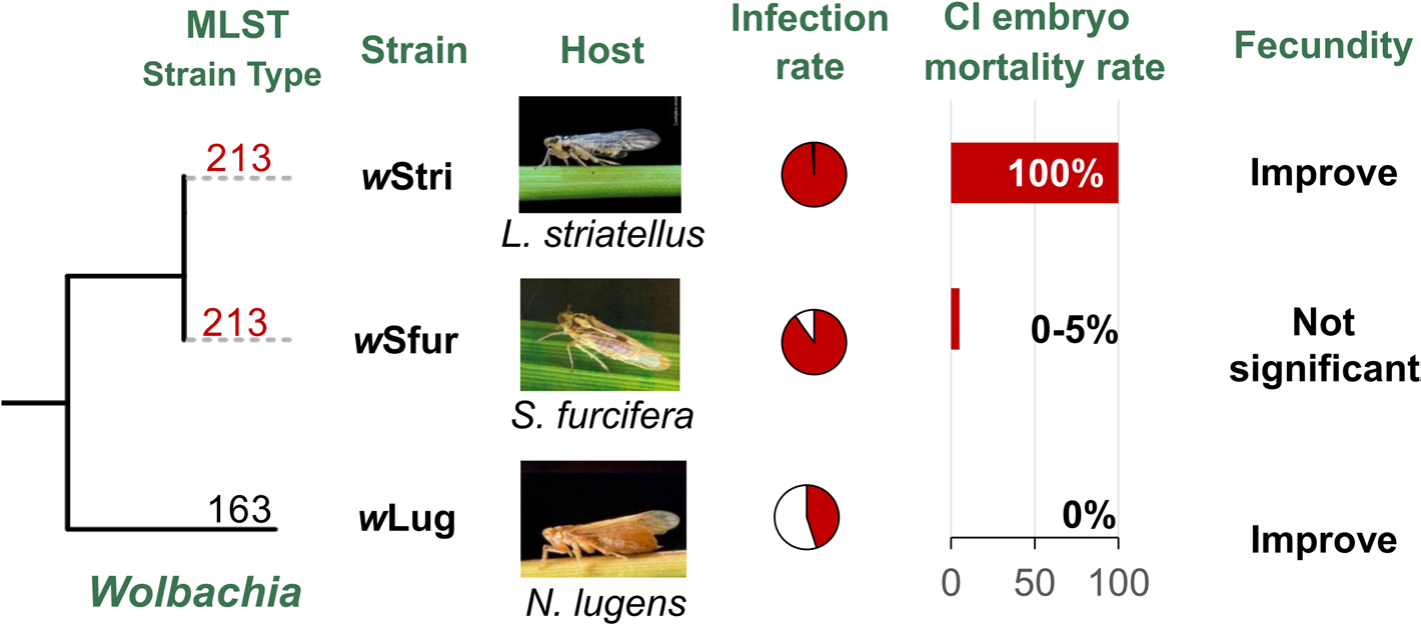
Reproductive effects of different *Wolbachia* strains on three planthopper species

Rice planthoppers harbor a variety of symbiotic bacteria, among which the endosymbiont *Wolbachia* is highly abundant in their hosts [9]. In *L. striatellus*, *Wolbachia* is the most abundant in all wild populations [10–12]. The infection of *Wolbachia* in planthopper populations appears to play a significant role in shaping the structure of microbial communities [12, 13], thereby influencing the distribution of the host. It significantly affects reproductive capacity and induces CI [14, 15]. Studies have shown significant variations in the *Wolbachia* strains infecting these planthoppers, including differences in infection rates, effects on host fecundity, and the ability to induce CI among three rice planthopper species [16–19] (Fig. 2).

The impact of *Wolbachia* on host reproductive capacity alters the base population size and is one of the factors influencing rice planthopper infestations. According to the CI principle, male insects carrying specific *Wolbachia* strains are released to mate with naturally uninfected females, causing fertilized eggs to die during embryonic development and effectively suppressing target pest populations [20]. This technique has been applied in mosquito control. In rice planthoppers, *w*Stri from *L. striatellus* has been successfully transfected into *N. lugens*, resulting in cytoplasmic incompatibility in the new host and the inhibition of rice ragged stunt virus infection and transmission, with significant pest control potential [21, 22]. Given the considerable agricultural damage caused by these planthoppers and the close relationship between their *Wolbachia* strains, cross-infection and colonization of different *Wolbachia* strains across species are possible. This presents an ideal model system for investigating the mechanisms through which *Wolbachia* regulates host reproduction.

### Bacterial factors associated with *Wolbachia*-induced cytoplasmic incompatibility

*Wolbachia* is transmitted mainly via maternal transmission. At the cytological level, although mature sperm do not contain *Wolbachia*, *Wolbachia* modifies or transforms sperm cells during the early stages of sperm maturation. This results in *Wolbachia*-infected (WI) males mating with *Wolbachia*-uninfected (WU) females. The resulting CI offspring, fertilized eggs, cannot effectively separate paternal chromosomes during cell division, forming a chromosome bridge and leading to embryonic death. However, *Wolbachia* within the eggs of WI females can "rescue" the paternal chromosomes, synchronizing the separation of maternal chromosomes and allowing normal embryonic development, a phenomenon known as “rescue” [23].

Because *Wolbachia* cannot be cultured freely in vitro, the key bacterial factors that induce CI have always been a challenge in studying its mechanisms. In recent years, with the development of sequencing technologies and advancements in research methods, the molecular mechanisms of CI have gradually emerged [8, 24]. Early studies revealed that the strength of the CI of *Culex* mosquitoes induced by *Wolbachia* was related to the genes encoding ankyrin (ANK) present in different strains [25]. In recent years, scientists have discovered that the proteins CifA (CI factor A, CidA or CinA) and CifB (CI factor B, CidB or CinB), which are encoded by two adjacent genes, may be key factors in the induction of host CI by *Wolbachia*. When WU male transgenic *D. melanogaster* expressing CifB and CifA mate with WU females, fertilized egg offspring exhibit the CI phenotype, and the embryos die. However, CifA and CifB can interact with each other. When WI male *Drosophila* infected with *Wolbachia* (*w*Mel strain) mate with WU females expressing CifA, fertilized egg offspring can develop normally, resulting in a "rescue" phenomenon [26–28]. Mutations in the PD-(D/E)XK nuclease, deubiquitinase DUB and CifA peptidase domains of CifB affect the function of CI factors [27, 29–31], indicating that the integrity of a specific domain is important for the normal function of CI. The three-dimensional structures of CifA and CifB have been resolved, and the interaction between the two proteins is related to charge attraction at three interfacial regions [32, 33].

**Fig. 3:**
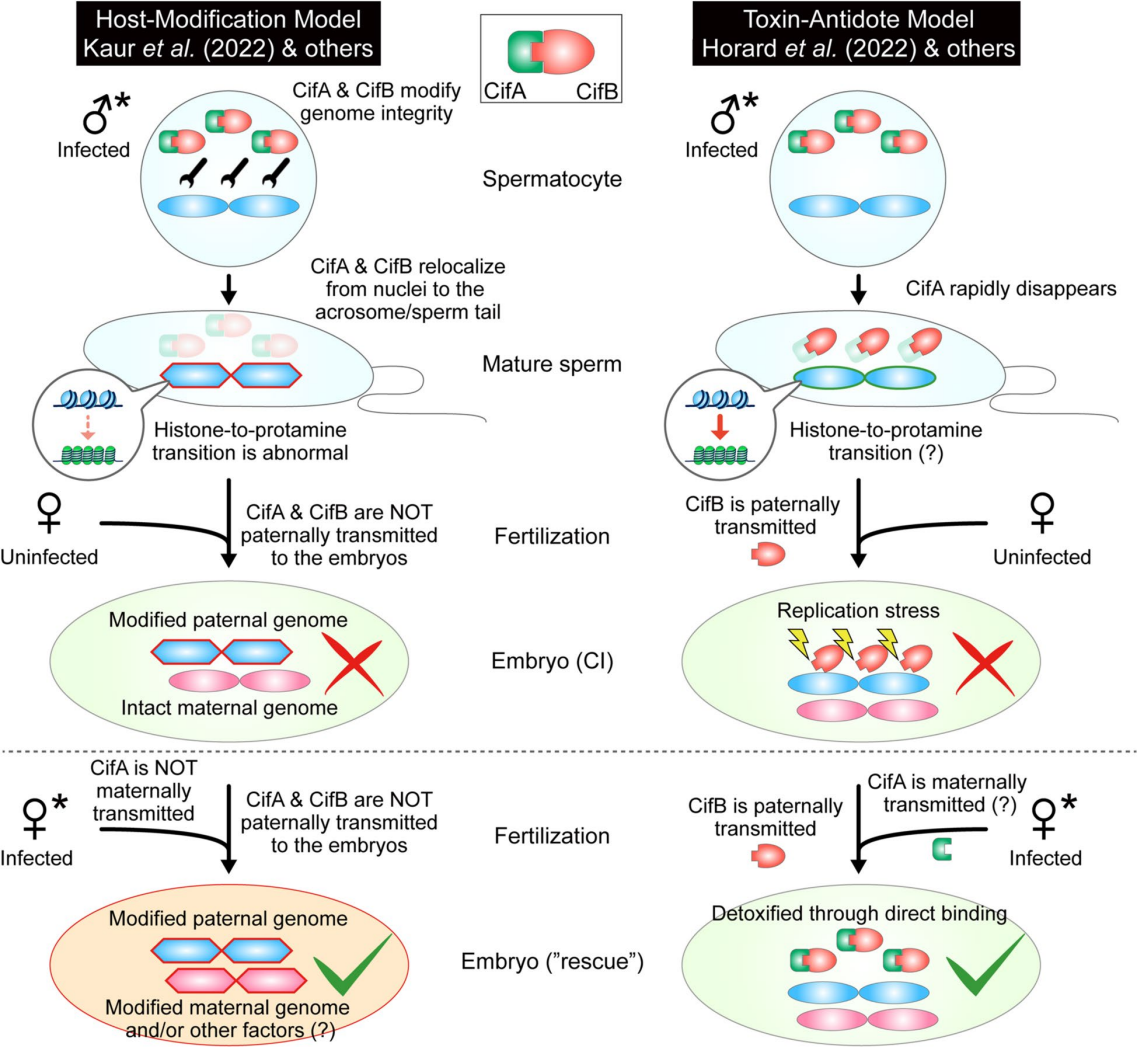
Two proposed mechanistic models for *Wolbachia*-mediated CI [35]

Although several studies have reported factors that induce CI in *Wolbachia* strains, Cif gene diversity is high, and evolutionary patterns are diverse [34]. The mechanism of CI is very complicated (Fig. 3) [35]. Currently, two models are used to explain the mechanism of CI, namely, the toxin-antidote model [36, 37] and the host modification model [38, 39].

The toxin–antidote model suggests that CifB is transferred to the embryo, where it is toxic. In the embryo, *Wolbachia* synthesizes CifA, which binds to CifB and neutralizes its toxic effects, thereby rescuing embryonic lethality. Both *w*Pip Cids are localized in developing spermatids until the histone-to-protamine transition, after which only CidB remains in the mature sperm. Additionally, CidB induces apoptosis in *Drosophila* S2R + cells, an effect that can be rescued by co-expressing CidA. CidB likely closely associates with paternal DNA to inhibit DNA replication, with replication resuming upon CidA binding to CidB [37].

The host modification model proposes that Cif proteins modify specific targets in gametes: sperm modifications induce CI, whereas female modifications rescue embryos by reversing these alterations to ensure normal hatching. Since CifB-mediated modifications occur only in developing sperm, CI neither requires CifB sperm transport nor CifA-CifB physical interaction [38]. For example, in *D. melanogaster* infected with the *Wolbachia w*Mel strain, CifA and CifB were detected in the sperm cell nucleus during spermatogenesis and sperm formation—this localization results in histone retention in elongating spermatids but protamine deficiency in mature sperm. In contrast, CifA was detected only in early-stage oocytes and was undetectable in mature oocytes or embryos, suggesting that CifA lacks the opportunity to bind CifB after fertilization for detoxification and is more likely to modify gamete DNA during gametogenesis. A similar Cif protein localization pattern has been confirmed in *Aedes aegypti* [39]. Functional studies further revealed that CifA, as a ribonuclease (RNase), degrades long non-coding RNAs (lncRNAs) involved in the histone-to-protamine transition during sperm formation. Moreover, both CifA and CifB act as deoxyribonucleases (DNases), exacerbating DNA damage in the late stages of sperm formation [40]. These molecular mechanistic findings collectively support the core hypotheses of the host modification model.

Most studies on the CI mechanism are still focused on mosquitoes and fruit flies, with some experimental results differing greatly between the two insects of the Diptera order [35, 41]. For example, CifA and CifB of *w*Mel from *D. melanogaster* need to be expressed simultaneously to induce the death of CI embryos [26, 40]. However, the expression of a single CifB protein from *w*Pip of *Culex* mosquitoes and* w*No of *Drosophila* was sufficient to induce CI [36, 42, 43], indicating that the functions of CI factors differ among different *Wolbachia* strains in different hosts.

The *Wolbachia w*Stri strain induces complete cytoplasmic incompatibility (CI) in its native host, *L. striatellus* [14]. When *N. lugens* is transfected into a new host, *N. lugens*, *w*Stri’s tissue distribution mirrors that of its native host, but its density is significantly higher than that of the *w*Lug strain. Although *w*Stri still induces CI in *N. lugens*, the intensity of CI is notably reduced [44]. These observations suggest that the strength of CI is influenced by both the symbiont and the host. Genomic analysis revealed that the *w*Stri genome contains three copies of the *cifA*-*cifB* genes, which are likely crucial for CI induction. Phylogenetic analysis revealed that the CI factor groups expanded from 5–10 groups, with *w*Stri factors belonging to newly identified groups VI and VIII [45, 46]. The functions of these CI factor candidate proteins remain unknown.

### Host factors associated with *Wolbachia*-induced cytoplasmic incompatibility

In host insects, studies in *D. melanogaster* have revealed that karyopherin-a, the protamine histidine chaperone P32, and ubiquitin-conjugating enzymes can interact with CifB, influencing CI lethality [47, 48]. The male histone-regulated gene *Hira*, the acyl-CoA-binding protein Acbp2 and the mitochondrial membrane protein Mcad may be involved in CI caused by *Wolbachia* infection [49, 50], but whether these proteins directly interact with CifA and CifB remains unclear. The modes of action of CI in different insects may differ. Therefore, the study of the CI phenomenon of *Wolbachia* in other organisms is particularly important for clarifying the action pattern of CI.

With the advantages of proteomics and RNAi technologies, Huang et al. reported that the cytoplasmic aminopeptidase-like protein (CAL) of *L. striatellus* might be associated with *Wolbachia*-induced CI death [51]. RNA-seq analysis revealed that *iLvE*, a gene involved in branched-chain amino acid (BCAA) biosynthesis, was the most significantly downregulated gene in WI *L. striatellus*. RNAi-mediated knockdown of *iLvE* in WU male *L. striatellus* resulted in reduced fertility and lower embryo hatching rates. However, fertility was restored when these males were crossed with *Wolbachia*-infected females, suggesting that *iLvE* may also contribute to the reproductive dysfunction associated with CI [52]. Another gene potentially involved in *Wolbachia*-induced CI in *L. striatellus* is *NDUFA8*, which encodes a subunit of the NADH dehydrogenase [ubiquinone] 1 α subcomplex. In WI females, the mRNA expression of *NDUFA8* was upregulated. RNAi-mediated suppression of *NDUFA8* significantly increased early embryo mortality without affecting the number of eggs laid. Furthermore, treatment with *dsNDUFA8* in WI females reproduced CI-like symptoms, suggesting that *NDUFA8* plays a key role in the rescue phenotype associated with CI [53]. These findings indicate that the interaction mechanisms between CI factors and the host may differ across different species. However, whether these proteins directly interact with CifA or CifB remains unclear. Moreover, functional validation of candidate CI and fecundity-related genes in planthoppers via techniques such as CRISPR/Cas9 or transgenesis would provide the most critical evidence.

### Mechanisms by which *Wolbachia* regulates host fecundity

*Wolbachia* infection can directly increase the fecundity of host insects. In *Aedes albopictus*, females infected with *Wolbachia* laid greater numbers of eggs [54]. *Wolbachia* is essential for host egg formation in *Asobara tabida* [55]. In *D. melanogaster*, *Wolbachia* manipulates the self-renewal and differentiation of reproductive stem cells to increase the fertility of the host [56]. *Wolbachia* can also provide nutrients to help the fruit fly *D. innubila* produce more female offspring under nutrient deficiency conditions[57]. The rice planthopper is a hemipteran insect that has undergone gradual metamorphosis and has significant biological differences from holometamorphic mosquitos, flies and wasps. The study of the function and mechanism of *Wolbachia* in other types of organisms is important for elucidating the interaction between *Wolbachia* and the host.

*Wolbachia* can also impact the fecundity of rice planthoppers. In *N. lugens*, the removal of *Wolbachia* results in a significant reduction in oviposition days and a decrease in the fecundity of female adults [58]. Similarly, *w*Stri significantly promotes the fecundity of *L. striatellus*. A previous study utilizing comparative genomics and metabolomics technologies reported that both *w*Stri and *w*Lug possess the complete ability to synthesize members of the vitamin B family, including biotin and riboflavin. These nutrients play crucial roles in maintaining the fecundity of both *L. striatellus* and *N. lugens* [59]. *Wolbachia*-provided riboflavin and biotin have been demonstrated to increase the fitness of the bed bug *Cimex lectularius* [60]. Moreover, biotin operons are found in *Wolbachia* genomes scattered across four supergroups [59, 61, 62], supporting the notion that the biotin operons were possibly laterally transferred from another organism.

At the host level, the increase in egg laying in the planthopper caused by *Wolbachia* was related to the upregulation of the expression of vitellogenin *Vg*, the increase in germ cell mitosis, and the induction of apoptosis in ovarian trophoblasts [63–65]. Previous studies suggest that *Wolbachia* accelerates nurse cells in female *L. striatellus* during peak oviposition, thereby providing more ovarian nutrients and increasing egg production during this period [65]. *Wolbachia* also affects miRNA expression in *L. striatellus*, thereby altering the expression of genes related to fecundity [66]. Recently, we reported that *Wolbachia* infection caused the differential expression of mitochondrial-related genes in planthoppers, suggesting that energy metabolism may also be related to *Wolbachia*’s improvement in the fecundity of planthoppers (unpublished data), but the underlying mechanisms remain to be further studied.

In *S. furcifera*, *Wolbachia* may interact with another intracellular endosymbiont, *Cardinium*. Different endosymbiont-infected lines present distinct microbiomes and metabolite levels, which influence their fecundity. Notably, *Cardinium* and *Wolbachia* double-infected lines present lower fecundity than uninfected lines do [67]. These findings suggest that the combined effects of *Wolbachia* and other symbionts may have a more pronounced impact on the host's reproductive capacity than either symbiont alone. Future research could explore the specific mechanisms underlying these interactions and their potential for manipulating pest populations through biological control strategies.

## Perspective

*Wolbachia*-mediated reproductive manipulation, particularly CI and fecundity enhancement, plays a pivotal role in shaping the population dynamics of rice planthoppers—among the most destructive pests in global rice production. The differential infection patterns, CI induction capacities, and reproductive benefits conferred by *Wolbachia* strains across *N. lugens*, *L. striatellus*, and *S. furcifera* highlight the complexity of symbiont–host interactions in hemipteran insects. Advances in comparative genomics, functional genetics, and proteomics have begun to unravel the molecular underpinnings of CI, identifying key effectors such as CifA and CifB and revealing potential host factors involved in modification and rescue pathways. Moreover, the role of *Wolbachia* in provisioning essential nutrients such as B vitamins and modulating host reproductive physiology offers new insights into mutualistic mechanisms that increase pest fitness. These findings not only deepen our understanding of insect–microbe coevolution but also open promising avenues for symbiont-based pest management strategies. Future research should focus on elucidating the host-specific determinants of CI strength, characterizing novel CI factor variants, and exploring the feasibility of leveraging *Wolbachia* for population suppression or replacement in rice planthopper control programs. Integrating *Wolbachia* biology into integrated pest management frameworks may offer sustainable, environmentally friendly solutions to mitigate the growing threat of planthopper outbreaks in rice agroecosystems. However, it is also essential to consider potential challenges in deploying *Wolbachia*-based control strategies in the field, such as the evolution of resistance or possible ecological side effects. However, the potential horizontal transmission of *Wolbachia* to non-target hosts must also be considered during risk assessments to avoid unintended consequences. In conclusion, while *Wolbachia* offers a promising avenue for controlling rice planthopper populations, the complexity of its interactions with both the host and the environment warrants careful consideration and further investigation.

## Data Availability

Not applicable.
